# Siloxane-functionalised surface patterns as templates for the ordered deposition of thin lamellar objects

**DOI:** 10.1038/s41598-019-54507-1

**Published:** 2019-11-29

**Authors:** Julian Hoffmann, Sofia Madrigal Gamboa, Andreas Hofmann, Hartmut Gliemann, Alexander Welle, Irene Wacker, Rasmus R. Schröder, Len Ness, Veit Hagenmeyer, Ulrich Gengenbach

**Affiliations:** 1 Institute for Automation and Applied Informatics, Karlsruhe Institute of Technology, Hermannvon-Helmholtz-Platz 1, 76344 Eggenstein-Leopoldshafen, Germany; 2 Institute of Functional Interfaces and Karlsruhe Nano Micro Facility (KNMF), Karlsruhe Institute of Technology, Hermann-von-Helmholtz-Platz 1, 76344 Eggenstein-Leopoldshafen, Germany; 3 Centre for Advanced Materials, Ruprecht-Karls-Universität Heidelberg, Im Neuenheimer Feld 225, 69120 Heidelberg, Germany; 4 Cryo Electron Microscopy, Universitätsklinik Heidelberg, BioQuant, Im Neuenheimer Feld 267, 69120 Heidelberg, Germany; 5 RMC Boeckeler, 4650 S. Butterfield Drive, Tucson, Arizona 85714 USA

**Keywords:** 3-D reconstruction, Electron microscopy, Surface assembly, Imaging techniques, Surface patterning

## Abstract

A novel method is demonstrated for ordered deposition of thin lamellar objects from a liquid environment onto solid substrates by solid/fluid/solid-driven organisation. Surface functionalisation forms a template pattern that accumulates the lamellar objects by site-selective wetting of the target area without the need for a physical fluid containment. Contrary to conventional handling methods, no mechanical contact occurs, which facilitates the ordered deposition without wrinkles or ruptures. An additive and a subtractive process for the creation of such templates are presented. The subtractive process starts with the complete silanisation of the substrate in the vapour phase followed by site-selective oxygen plasma treatment of the siloxane film. The additive process uses microcontact printing to transfer the target pattern. Both processes are characterised by optical inspection of the wetting contours and it is found that site-selective plasma treatment shows a better pattern fidelity. The patterns obtained by site-selective plasma treatment are also subject to ToF-SIMS analysis and show good chemical contrast between hydrophilic and hydrophobic areas. The ordered deposition of lamellar objects by this new method is demonstrated for 60 nm thick ultramicrotome sections of epoxide resin on pre-patterned glass substrates.

## Introduction

In many applications, the handling of thin lamellar objects with extreme aspect ratios spanning multiple length scales is a technical challenge. Surface and line forces become dominant in these realms and the object’s shape provides almost no stiffness, rendering destruction-free handling difficult. Particularly sensitive nanometer-thin lamellar objects are encountered in thin film technology or ultramicrotome sectioning.

In thin film technology, it may be necessary to transfer thin films from a supporting substrate, which has been used for film preparation, to a different target substrate. Floating-off methods are established procedures in this field. One example is the application of conjugated microporous polymer (CMP) films as membranes for gases, where the CMP was originally produced on a solid substrate and is afterwards transferred to porous carbon nanomembranes to investigate the gas filtering properties of the CMP film^1^. Moreover, floating-off of thin films is used as a method for coating technical devices by film transfer in case the devices do not withstand the conditions of a direct film preparation^2^. Although the floating-off of the thin films is usually unproblematic, picking up the floating film might be challenging, as e.g. under mechanical stress wrinkling or even rupture of the film may occur^3,4^.

Another typical challenge is the transfer of ultramicrotome sections on solid substrates for imaging purposes. Serial ultramicrotome sectioning is a sample preparation technique for investigating the 3D nanostructure of biological (e.g. cells) or technical specimens (e.g. printed electronic devices) by means of light or electron microscopy. Cross-sections are obtained by cutting a specimen embedded in a resin with a diamond knife into ultra-thin sections, with a typical thickness of 30 nm–200 nm and lateral dimensions of 0.2 mm–1 mm. During the cutting process, the sections are pushed onto the surface of a water reservoir in the knife boat and form section ribbons. In order to make these sections available for imaging, they must be transferred onto a glass or silicon substrate undamaged (e.g. without wrinkles) and in well-defined order. To date, by means of makeshift tools (typically an eyelash attached to a toothpick), a skilled operator can manually transfer small numbers of sections onto a substrate submerged in the water reservoir. For large quantities of sections e.g. in Array Tomography (AT) and Correlative Array Tomography (CAT), this is no longer feasible^5,6^. Hence, dedicated means to automate this process must be developed^5,6^. The sensitivity of the fragile sections makes the application of mechanical methods for automated section handling difficult.

In both thin film technology and ultramicrotome sectioning, lamellar objects are floating in a liquid environment to facilitate deposition on solid substrates. This process environment however, a solid substrate whose surface properties can be tailored and which is submerged in a liquid reservoir, opens up interesting possibilities for ordered deposition of floating lamellar objects by solid/fluid/solid interface-driven organisation.

The wettability of a substrate surface depends on its surface roughness and its surface free energy (SFE). The SFE is an important property which can be modified e.g. by chemical functionalisation to tailor the surface interaction with organic, inorganic or biological materials to the specific application. Depending on the task, a substrate surface is partly or entirely functionalised to create application-specific patterns. Examples are surfaces consisting of tripeptide Arg-Gly-Asp (RGD) motive functionalised patterns on glass^7^ or UV light-induced patterned polymer surfaces^8^ to control the adhesion of eukaryotic cells. Apart from solid-solid interactions, interactions between a patterned solid substrate and liquids are also reported. One application for such patterns is the self-aligned deposition of fluids. An example is the high throughput analysis in droplet microarrays, where discontinuous dewetting fills individual wells without mechanical containment^9,10^. In printed electronics, the deposition of functional inks constrained by hydrophobic barriers allows the creation of smaller structures^11^ and the tailoring of surface properties to the requirements of different inks^12^. In technical applications, surface functionalisation has been used to create hydrophilic binding site patterns for the self-assembly of sub-millimeter silicon chips onto hydrophobic substrates submerged in water^13^.

One widely applied method to chemically modify the properties of oxidic or oxide-like substrate surfaces is silanisation, where a molecular bond is formed by hydrolysis of a silane/siloxane and condensation with an OH-group on the substrate surface. This technique is used to form self-assembled monolayers (SAMs) or thin films with molecular thickness^14^.

In the present paper 1,7-Dichloro-octamethyltetrasiloxane is used to create durable hydrophobic thin films with polydimethylsiloxane (PDMS)-like material properties. The methyl termination is responsible for the chemical inertness and low surface energy. Silanisation is typically performed by immersion in a siloxane/solvent mixture in the liquid phase, resulting in coatings of 2 nm thickness with contact angles (CA) above 100°^15^. Silanisation with similar siloxanes in vapour phase has also been demonstrated^16^.

Patterns can be created either in subtractive or additive processes. Subtractive approaches facilitate silanisation of the entire substrate surface by liquid or vapour phase deposition and subsequent patterning, e.g. by plasma- or laser-based processing^17^. Additive approaches directly form the patterns on a substrate with inked stamps, e.g. microcontact printing^18,19^.

**Figure 1:**
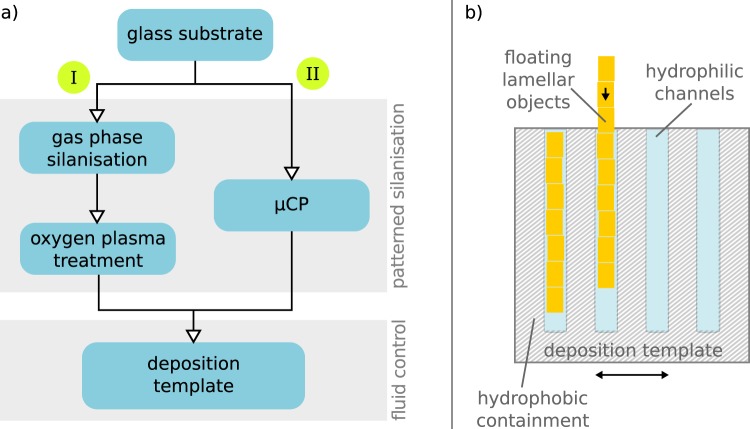
(**a**) Proposed process for subtractive (I) and additive (II) manufacturing of functionalised templates, (**b**) Schematic of the ordered deposition of thin lamellar objects on templates with hydrophilic channels and hydrophobic areas to contain the liquid environment on the target substrate.

In order to create functionalised surface patterns, we compare an additive and a subtractive process, as illustrated in Fig. 1, to obtain a well-defined difference in the SFE between pristine glass and siloxane coated areas. A glass surface has a high SFE after an oxygen plasma treatment with polar Si-OH groups while the nonpolar methyl termination of a siloxane coating has a very low SFE. The subtractive process is based on gas phase silanisation to form a thin hydrophobic siloxane coating followed by site-selective oxygen plasma-induced decomposition of the siloxane in unmasked regions to create hydrophilic areas. We select the deposition in vapour phase to perform the silanisation with a minimal amount of chlorosiloxane exposed to ambient humidity. Furthermore, unwanted growth of agglomerations, which has been observed after complete immersion in liquid phase, should be avoided^20^. On the contrary, the additive process starts with an oxygen plasma-treated hydrophilic substrate and adds a patterned hydrophobic siloxane layer by micro contact printing (µCP) with a chlorosiloxane inked stamp. Through a sharp contrast between hydrophilic plasma-activated glass surface and hydrophobic siloxane coated surface, we expect a well-patterned and defined difference in the SFE.

Two approaches for the creation of patterned hydrophobic/hydrophilic surfaces by siloxane surface modification are presented. The most suitable process is selected to create templates that facilitate the ordered deposition of 60 nm thin ultramicrotome sections onto solid substrates as a promising use case for the ordered deposition of thin lamellar objects. The surface modification controls spreading of a water film, which itself is the carrier for floating lamellar objects. The presented handling method demonstrates how selective surface modification can immobilise sensitive lamellar objects with nanometer thickness by solid/fluid/solid interface interactions. The direct manipulation of the surface free energy allows finer patterns compared to a mechanical containment of the wetting fluid and avoids physical contact. Multiple parallel narrow hydrophilic channel templates allow the deposition of lamellar objects in a dense packing while also preserving their order. The scientific novelty is the application of a chemically-tailored solid/fluid/solid interface-driven organisation for the manipulation of floating thin lamellar objects, which is still a challenge^4^.

## Methods

### Substrate preparation

75 mm × 25 mm microscopy slides (631-0113, VWR, Germany) are used for the evaluation of the patterning method and smaller 22 mm × 22 mm microscope coverslips (470055, Brand, Germany) as substrates for the deposition of ultra-thin sections. Before the experiments, the substrates are soaked in cleaning solution (2% Micro-90, Sigma-Aldrich, Germany) for 24 h and rinsed thoroughly with de-ionised (DI) water, followed by 10 min ultrasonic cleaning in acetone and isopropyl alcohol respectively. Subsequently, the substrates are rinsed with DI water, oven-dried for 2 h at 120 °C and stored in sealed containers. Immediately before the silanisation processes, the substrates are treated in an oxygen plasma (Diener Atto, Germany) for 10 min at 0.5 mbar to increase the number of available OH-surface groups for siloxane bonding.

### PDMS stamp fabrication

**Figure 2:**
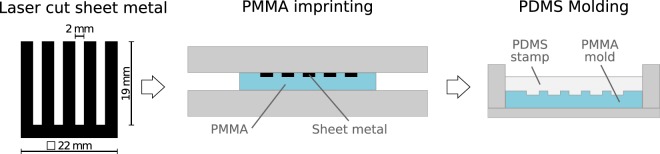
Laser-cut sheet metal used for PMMA imprinting to create PMMA moulds to cast PDMS stamps.

Figure 2 illustrates the stamp preparation process. Laser-cut stainless steel sheet metal (size of 22 mm × 22 mm, thickness of 200 µm, PCB mask manufacturer, Beta LAYOUT, Germany), with the shape of the target pattern, are used as masters for hot embossing. The sheet metal is placed on polymethylmethacrylat (PMMA) sheets (Plexiglas, Evonik Industries, Germany) with 2 mm thickness and hot-embossed with an applied force of 2.2 kN in a mechanical press at 125 °C for 1 h. The PMMA mould is then cooled down to 80 °C within an hour and annealed at that temperature for another hour to relieve stress. To facilitate later demoulding of the final stamp, the resulting PMMA mould is treated with perfluorodecyltrichlorosilane (FDTS) (97%, Alfa Aesar, Germany). Poly(dimethylsiloxane)-based (PDMS) stamps (Sylgard 184® elastomer, Dow, Germany) are prepared by mixing elastomer and curing agent in a 10:1 weight ratio and cured at 75 °C for 2 h in the PMMA moulds. After demoulding the PDMS is rinsed with heptane to remove excess volatile components and dried in air.

### Siloxane patterning of the substrate

#### Method I: subtractive patterning

Vapour phase silanisation: A 5% solution of 1,7-dichloro-octamethyltetrasiloxane (Cl[Si(CH_3_)_2_O]_3_Si(CH_3_)_2_Cl) in heptane (Sigmacote®, Sigma-Aldrich, Germany) is used for the preparation of hydrophobic coating. Complete silanisation of the substrate surface is performed by arranging 10 microscope slides vertically with 10 mm spacing in a sealed container with a volume of 900 cm^3^. The container is placed in an oven preheated to 55 °C to allow homogeneous heat distribution and then 150 µl of siloxane (5%) is added to perform vapor phase silanisation for 60 min at 55 °C. Subsequently, the substrates are rinsed with DI water.

**Figure 3:**
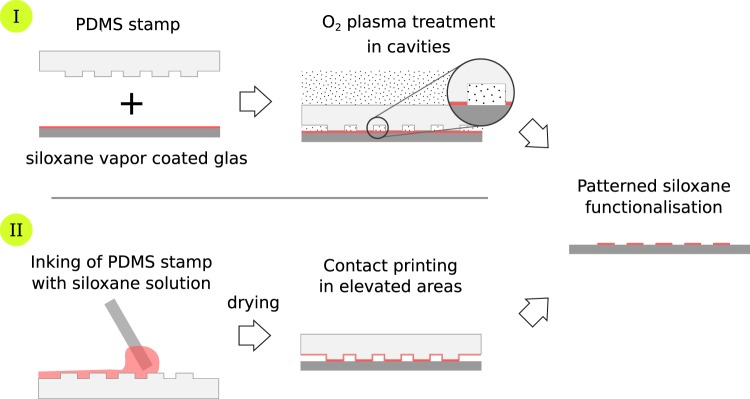
Process steps of oxygen plasma treatment in cavities (I) and microcontact printing (II) to create a patterned siloxane modification as a deposition template.

Oxygen plasma patterning: The silanised surface is then covered with the PDMS stamps and exposed to an 13.56 MHz RF oxygen plasma at 0.5 mbar (Diener Atto, Germany) for 15 minutes. The stamp protects the siloxane layer at the contact surfaces and allows plasma treatment of the layer in the 200 µm high cavities as illustrated in Fig. 3.

#### Method II: additive patterning

Microcontact printing: 1,7-dichloro-octamethyltetrasiloxane (95%, Sigma-Aldrich, Germany) is used for microcontact printing experiments. The PDMS stamps with an outer dimension of 22 mm × 22 mm are inked with a freshly prepared solution of 10% chlorosiloxane in acetone (p. a., Merck, Germany) as illustrated in Fig. 3. The stamps are air-dried for 20 s and then brought into contact with the glass surface for 60 s. Subsequently, the substrate is rinsed with DI water.

### Pattern characterisation

#### Contact angle analysis

The hydrophobicity of siloxane coatings created by vapour phase silanisation is characterized with a drop shape analyzer (DSA 100, KRÜSS, Germany) by measuring the mean static contact angle (CA) between distilled water and the substrate surface. Two measurements using the sessile drop method are performed on each substrate surface. For the measurement, a droplet volume of 10 µl is dispensed and the resulting contour fit to a Young-Laplace model for contact angles >10° and to a circle model for smaller contact angles. The baseline is set manually and measurements are done 10 s, 30 s, 1 min and 2 min after drop deposition.

#### Characterisation of the fluid containment properties of the channel templates

In order to assess the fluid containment properties of the resulting channel templates, the hydrophilic channels are filled with an increasing amount of water and inspected optically. The fluid inlet is placed close to the beginning of the channels and slowly (10 µl/min) filled with DI water using a syringe pump (NE-1000, New Era Pump Systems Inc, USA). Images are taken at 0.88 µm resolution with an optical microscope (INM 200, Leica, Germany). Using the refraction at the water surface in top illumination, spreading of the wetting fluid into the wettable channel area is clearly visible, allowing automated analysis with image processing tools (canny edge detection algorithm, scikit-image library). The contour of the spread fluid is fit against a straight nominal shape to estimate the length and width of the hydrophilic channels. The root of the quadratic fitting error (standard deviation) is used to estimate the deviation from the nominal shape. The channels are then filled with water until the fitting error increases steeply, which indicates the maximum water storage capacity.

#### ToF-SIMS surface analysis

Time-of-Flight Secondary Ion Mass Spectrometry (ToF-SIMS) is performed in a ToF.SIMS5-100 (ION-TOF GmbH, Germany). Ultra-High Vacuum (UHV) base pressure is <5 × 10^−9^ mbar. For high mass resolution the Bi source is operated in a bunched mode providing short ${{\rm{Bi}}}_{3}^{+}$ primary ion pulses at 25 keV energy and a lateral resolution of approx. 4 µm. The short pulse length of 1.5 ns allows high mass resolution. Charge compensation during spectrometry is necessary due to the highly insulating nature of the glass substrates. Therefore, an electron flood gun providing electrons of 21 eV is applied, and the secondary ion reflectron tuned accordingly. Images with a field of view of several square millimeters are obtained by scanning the sample stage and are recorded with a 10 µm pixel size. For high lateral resolution imaging at the channel edge, in order to determine the patterning fidelity, a non-bunched primary ion mode is used. With nominal mass resolution the signals of SiO_2_, SiO_3_, and SiO_3_H are recorded with 256 × 256 pixel in a field of view of 500 µm × 500 µm. The pattern fidelity at the channel edge is obtained by adding the glass and siloxane signals parallel to the edge.

#### Deposition of ultramicrotome sections

Blocks of epoxide resin are prepared using a two-component epoxide embedding kit (EpoFix kit, Science Services, Germany) with a volume ratio 15: 2 of resin to hardener and cured for 2 days at room temperature. The blocks are trimmed to a typical slightly trapezoidal shape with 0.85 mm width on the short side and 1.4 mm height using a 90° diamond trimming knife (Trim 90, Diatome, Switzerland). The block is then cut into 60-nm thin sections using an ultramicrotome (Powertome XL, RMC, USA) and a modified diamond knife (Ultra Jumbo, Diatome, Switzerland) with an extra wide boat to allow lateral movement of the substrate across the complete length of the diamond knife. Sectioning is performed with standard settings as recommended by the knife manufacturer. The substrates are patterned according to the subtractive process by plasma-induced siloxane degradation and submerged into the DI water-filled reservoir of the modified diamond knife boat. Using a motorised 3-axis handling system, the substrates are then lifted to allow dewetting in the hydrophobic containment areas and the formation of a thin water film on the hydrophilic channels. Afterwards the channels of the template are aligned with the section ribbon and further sectioning is performed to push the section ribbon inside the channel. When a channel is filled with sections, the section ribbon is separated manually, and the substrate moved laterally to align the next empty channel with the end of the section ribbon. The process of cutting, separation and substrate shifting is then repeated to fill the remaining channels with sections. After all channels are filled, the final deposition of the sections on the substrate is accomplished by slowly lifting the substrate out of the water reservoir and allowing the remaining water to air dry.

## Results and Discussion

### Vapour phase silanisation

**Figure 4:**
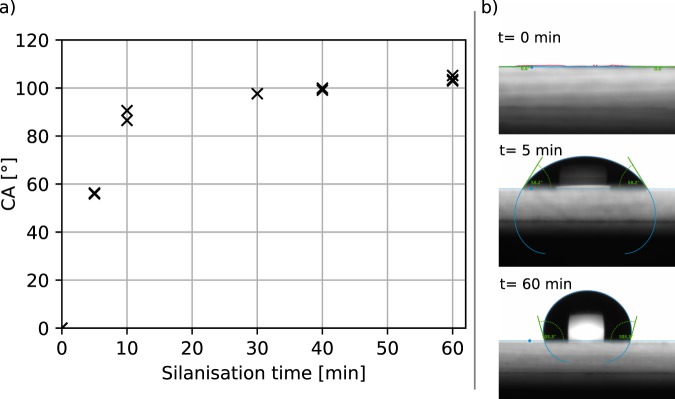
(**a**) Measured mean contact angle (CA) (n = 8, µ < 2°) for 12 glass slides undergoing different silanisation times in 1,7-Dichloro-octamethyltetrasiloxane vapor, (**b**) Representative drop shapes after 0 min, 5 min and 60 min of silanisation.

The hydrophobicity of glass substrates silanised with 1,7-Dichloro-octamethyltetrasiloxane vapour is investigated by measuring the static contact angle after different silanisation times. As shown in Fig. 4, the CA increases rapidly in the first 10 min of exposure, while it levels off from 10 min to 60 min. At 60 min, the CA reaches a value of 105° which is comparable to literature values of 110° for the immersion in chlorosiloxane solution^15^. The results indicate that a hydrophobic functionalisation (>90°) is clearly achieved after 60 min of exposure. The resulting functionalisation is uniform with a standard deviation (µ) of the measured CA less than 2° over two measurement points on the sample surface and four measurements per measurement point for each substrate. The contrast in wettability compared with an oxygen plasma-treated substrate surface is sufficient to allow containment of the wetting fluid. A silanisation time of 60 min is selected for patterning by oxygen plasma-induced siloxane decomposition. Silanisation is performed with 150 µl of chlorosiloxane solution which is considerably below the amount required for immersion in the liquid phase. The potential influence of contamination of the chlorosiloxane solution through reaction byproducts during silanisation as by immersion of the substrates is avoided^20^. The demonstrated batch-like parallel silanisation of 10 substrates requires minimal actions by the operator. The necessary equipment is limited to a sealed containment and an oven and allows the process to be easily replicated in other laboratories with standard equipment. The low deviation (µ < 2.5°) of the resulting mean CA over four repeated experiments for 60 min of silanisation indicates that the process also provides a functionalisation with reproducible properties. A homogeneously-silanised glass slide is also subjected to ToF-SIMS analysis on different length scales. For defect detection several images are recorded down to a set of 3 × 3 images with 100 µm × 100 µm field of view and 512 × 512 pixel resolution. In these images, no defects larger than 2 µm^2^ are found within an inspected area of 90.000 µm^2^ (recorded images in Supplementary Data S7).

### Pattern quality

The two processes presented, following either an additive or a subtractive approach, create functionalised surface templates that are capable of containing water in hydrophilic channels. Both processes maintain the edge profile along the complete length of the channels. For oxygen plasma patterning, the pressure and exposure time are adjusted to facilitate treatment over the full length of the cavities of the PDMS stamp. For microcontact printing, the printing time and drying time are varied. Neither process requires extensive preparation and can be used to pre-pattern the substrate with a deposition template before the deposition of lamellar objects. The process of microcontact printing involves steps that require precise performance in order to achieve consistent results. Especially the timing of the drying and printing process steps can be a challenge, ambient humidity may contribute an additional uncertainty to the process and a significant technical effort is required to control all preparation parameters of the environment where the printing process is carried out by the operator (e.g. glovebox). It is observed that the silanisation solution does not have a long shelf time as it starts to become turbid very quickly and is unusable within less than 24 h. This indicates that the chlorosiloxane polymerises in the solution. In contrast, the steps during the patterning by oxygen plasma treatment do not require as much technical effort and vapour phase silanisation can be carried out in small containers with less ambient influences.

**Figure 5:**
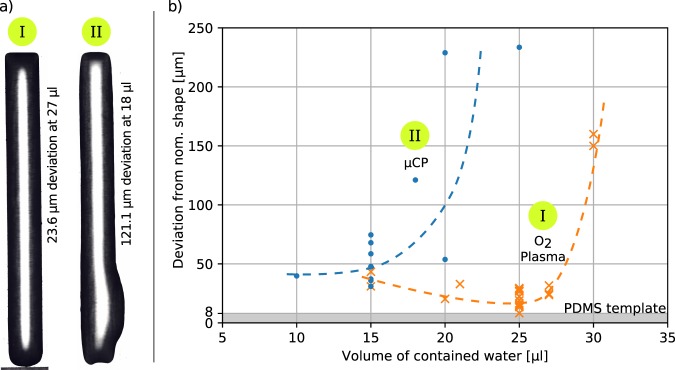
(**a**) Representative top view of wetted channels through subtractive (I) and additive (II) patterning. Note the imperfect channel geometry in (II) at a smaller volume of contained water than (I), (**b**) Plot of measured edge deviation from nominal shape for channels created by subtractive (n = 13) and additive patterning (n = 8) at different volumes of contained water. Dotted lines show qualitative trends.

Optical inspection of the channel-filling process shows that the subtractive process allows a higher amount of water to be contained before the fluid breaks out of the hydrophobic containment (Fig. 5a). A smaller deviation of the wetted pattern from the straight target shape will likely result in less edge imperfections that obstruct section movement while sliding down the channel. This can be seen in Fig. 5(b) as the edge deviation for templates created by oxygen plasma-induced patterning (orange crosses) stays closer to the reference value of the 8 µm edge deviation of the PDMS stamp as compared with the templates created by microcontact printing (blue dots). The edge deviation does not increase noticeably as long as the volume of contained water is <30 µl for templates created by plasma-induced patterning. For templates created by microcontact printing, the deviation already significantly increases for volumes >20 µl. This indicates that the subtractive process is able to create patterns with a higher pattern fidelity and well-defined difference in the SFE.

**Figure 6:**
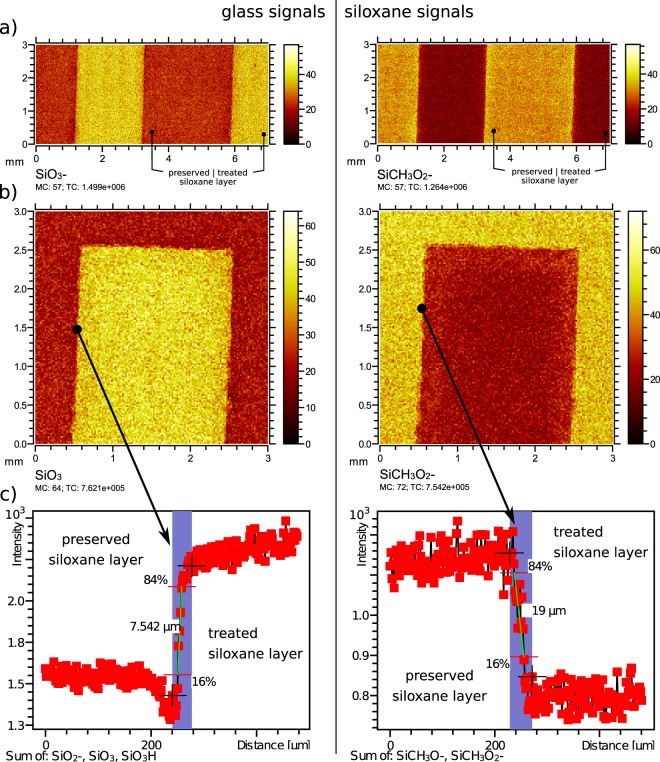
ToF-SIMS images of channels patterned by site-selective oxygen plasma treatment. ${{\rm{SiO}}}_{3}^{-}$ indicates predominantly glass (left), while SiCH_3_${{\rm{O}}}_{2}^{-}$ indicates the siloxane layer in between the channels (right). (**a**) Large field of view (3 mm × 7 mm) showing two adjacent channels, (**b**) ToF-SIMS imaging of a channel end, (**c**) Intensity line profile added over 0.5 mm length along a single channel edge with high lateral resolution mode.

To further support the finding that samples created by plasma-induced patterning led to a well-defined transition in the SFE, an analysis of the chemical composition of the surface patterns by ToF-SIMS is performed. Figure 6 shows the measured intensities for the negatively charged fragments associated with the siloxane layer (SiCH_3_O_2_) and the glass surface (SiO_3_). The results support the statement that a well-defined SFE transtion is the result of the well-defined siloxane patterning. The siloxane in the hydrophilic areas is homogeneously decomposed by the oxygen plasma. It is possible that the siloxane in the hydrophobic areas partly originates from the PDMS stamp. A differentiation between ion fragments originating from the siloxane coating or a diffusion from the PDMS stamp is not possible using ToF-SIMS as the two compounds are too similar. It is visible that the signals for the fragments associated with siloxane (SiCH_3_O and SiCH_3_O_2_) do not drop to near zero in the plasma-treated areas. This is due to the plasma-induced decomposition of the siloxane leaving residual fragments. While those fragments are still visible in the ToF-SIMS measurements, they will have a much lower SFE than the long siloxane chains with their unpolar methyl end groups as the wetting analysis in Fig. 5 shows. Dynamic ToF-SIMS measurements as shown in Supplementary Data S1–S4 provide a higher intensity ratio of 1:4 compared with a 2:3 ratio in static ToF-SIMS measurements displayed in Fig. 6. After 12 months of storage, the steepness of the transition of the siloxane signals as measured in Fig. 6(c), Supplementary Data S1 and S4 has not noticeably decreased. From this, we conclude that the siloxane layer must be stable.

As shown in Fig. 6(b) the channel is fully plasma-treated up to the end of the cavities formed by the applied PDMS stamp. As shown in Fig. 6(c), according to a 84–16% intensity change definition^21^, the transition length is smaller than 20 µm for the siloxane signal and smaller than 8 µm for the glass signal over an analysed length of 0.5 mm. Several effects contribute to the patterning fidelity: defects in the edge of the cast PDMS stamp that is used, sagging of the PDMS elastomer when pressed onto the glass (long-range bending), edge effects during the plasma treatment, migration of low molecular weight siloxanes from the PDMS onto the glass surface, multiple contacts of the stamp during the manual positioning or movements of the PDMS stamp during plasma treatment. The lateral resolution of the applied ToF-SIMS imaging setup in Fig. 6(c) is better than 1 µm and sufficiently below the measured transition lengths from glass to siloxane layer. The pattern fidelity at the channel edge corresponds well with the measured 8 µm deviation of the stamp edge. A mechanical device as illustrated in Supplementary Data S6 is used for positioning the PDMS stamp, minimising positioning errors, ensuring a uniform contact pressure and high reproducibility. The device has a positioning tolerance of ±10 µm. Comparing the aforementioned contributing effects, the geometry and fidelity of the stamp are the limiting factors under the constraint that great care is taken during the positioning of the stamp on the substrate prior to plasma treatment by using the described positioning device.

Pre-patterning of the substrate with a deposition template is preferably performed using site-selective plasma treatment because of its superior properties to contain water in the desired target shape and feasibility of the process steps.

### Ordered deposition

**Figure 7:**
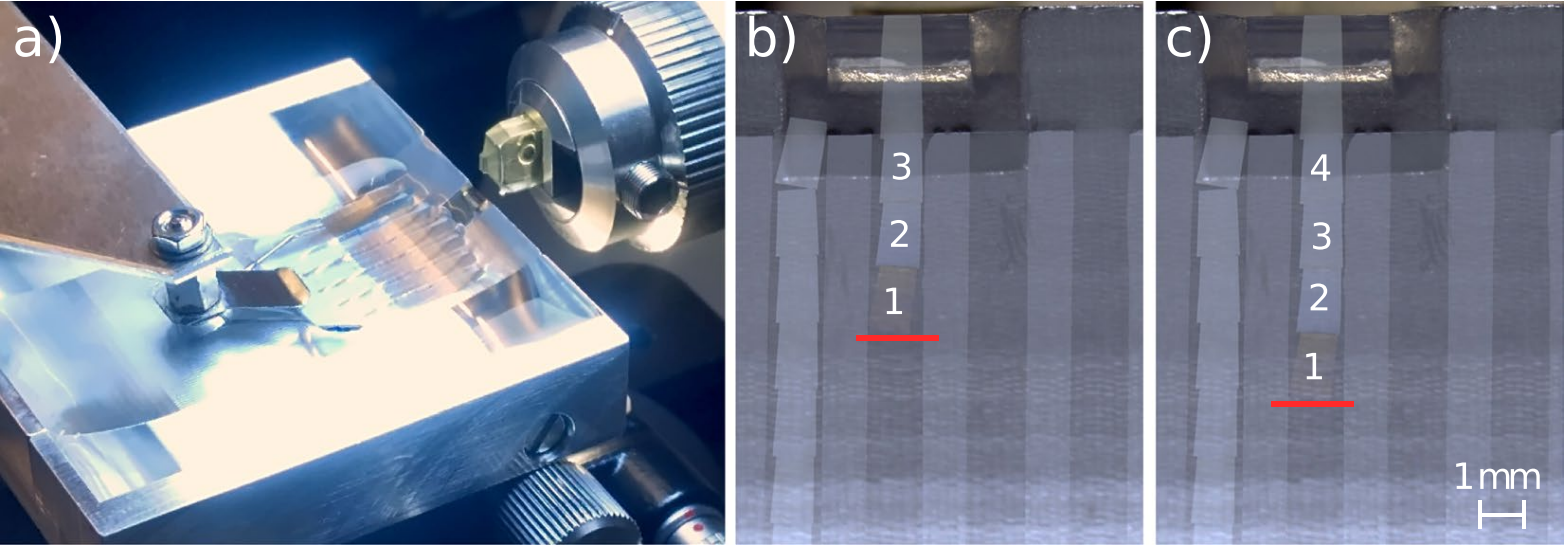
(**a**) Ultramicrotome setup with pre-patterend substrate submerged in a modified extra wide boat, (**b**) Top view of the substrate with 60 nm (2, 3, 4) and 100 nm (1) thick lamellar sections sliding on a water film inside hydrophilic channels onto the glass substrate and (**c**) after adding an additional section (4). The red mark indicates the section ribbon being pushed down the water-filled channel (see Supplementary Video S8).

The ordered deposition of lamellar objects by pre-patterned templates is shown for 60 nm thin ultramicrotome sections. The jamming of ribbons can be avoided by providing more guidance to the sections. Preferably the cutting parameters are adjusted such that the sections form straight ribbons. Imperfect trimming of the sample block or inhomogeneous compression due to sample properties may cause curved ribbons. Thus, curved ribbons cannot entirely be avoided and must also be accommodated by the channels. An individual rectangular section with 1.4 mm length and 0.85 mm width can in principle rotate completely inside a 2 mm wide channel. It has been observed that if the first section encounters the boundary not tangentially but at a rather steep angle (>15°), the sections tend to become stuck and fold. A shallow (<15°), ideally tangential encounter between the first section of a ribbon and the boundary guides the sections inside the channel without damage. As a consequence the channel width was reduced to 1.2 mm. This limits the possible angles at which the sections can encounter the boundary to be <15°. The calculation and values for the reported section shape can be found in Supplementary Data S5. At small angles of encounter, the complete filling of multiple channels with sections is possible, as seen in Fig. 7. The amount of water wetting the channels can be controlled by the immersion depth of the substrates inside the water reservoir of the knife boat. A minimal amount of water wetting the hydrophilic areas shows best results, because of a flat meniscus in the channel centre. A high meniscus is, however, necessary at the front end of the channel, where the water film connects with the meniscus behind the knife edge. The goal of the alignment between channel and knife edge is to provide a path with low surface curvature which guides the sections into the channels. Typically, an operator will try to align five section ribbons onto a substrate by conventional manual manipulation, which requires skill and training. In contrast, the template with 1.2 mm wide channels on a 22 mm × 22 mm substrate (Fig. 7) allows 10 section ribbons to be aligned with the lateral positioning performed by a motorised handling unit. Since only a thin water film forms on the template, the sections are effectively shielded from turbulence inside the water reservoir of the knife boat e.g. caused by a lateral movement of the substrate. This way switching to the next empty channel is possible without the requirement to pin the sections in already filled channels. The pushing force of the cutting process is sufficient to move the sections into the channel as the sections slide along the channel edges on a thin water film. It is possible to fill a 19 mm long channel over its complete length without the sections starting to fold due to interaction with the channel boundary. The sliding process requires that the channel boundary does not contain imperfections as this would obstruct section movement. Retrieval of the completed, section-filled substrate is done by slowly lifting the substrate from the knife boat using an automated 3-axis handling system. Since it is known that treatment with air (oxygen) plasma helps to avoid the formation of folds in the sections^22^, during their drying after removal from the water reservoir we surmise that the hydrophilicity of the channels allows a smooth retraction of the water meniscus and a settling of the lamellar objects without folds or wrinkles. The resulting pattern of the deposited sections follows a straight alignment in rows and preserves the section order by storing them in separated, pre-selected channels. This way the order and orientation of sections is easily traceable which is a requirement e.g. for Array Tomography.

## Conclusion

Two processes patterning templates on glass substrates for the ordered deposition of thin lamellar objects by solid/fluid/solid interface-driven organisation are presented. Silanisation with 1,7-Dichloro-octamethyltetrasiloxane is used to create a hydrophobic surface functionalisation. The surface functionalisation is patterned either in an additive or a subtractive process to create a template with parallel hydrophobic channels having a dead end. The surface properties i.e. hydrophobic contrast, are assessed by contact angle and ToF-SIMS measurements. Moreover, shape and water containment properties of the resulting channels are measured by image processing. The subtractive process by oxygen plasma-induced decomposition of the siloxane coating shows superior results. The process requires only basic equipment and can easily be performed by qualified lab staff. Templates patterned by this process are used for the aligned deposition of ultramicrotome sections. The experiments indicate that this is a promising handling method for densely ordered deposition of such lamellar objects in rows on a glass substrate and has the potential to be automated. Although only deposition on glass is demonstrated here, it is likely that the method can be adapted to other solid oxidic substrates such as ITO-coated glass or silicon wafer. Hence this method has the potential to become a valuable tool for sample preparation in Array Tomography and Correlative Array Tomography. Moreover, it can be applied to the ordered transfer of other types of thin lamellar objects onto substrates e.g. created by float-off methods in thin film technology.

## Supplementary information

Supplementary Data S1-S7

Supplementary Movie S8

## Data Availability

ToF-SIMS images of defect inspection and a video of section deposition can be found in the Supplementary Information. The complete generated ToF-SIMS data sets are available from the corresponding author on reasonable request.
