# Supplementary material for: Siloxane-functionalised surface patterns as templates for the ordered deposition of thin lamellar objects
Source: Sci Rep. 2019 Nov 29;9:17952. doi: 10.1038/s41598-019-54507-1 (PMC6884528; doi:10.1038/s41598-019-54507-1)
Supplement: Supplementary file 1 — Supplementary Data S1-S7 [file 41598_2019_54507_MOESM1_ESM.pdf]

Siloxane-functionalized surface patterns as templates for the ordered deposition of thin lamellar objects - Supplementary Data S1-S7

Julian Hoffmann *[,1], *Sofia Madrigal Gamboa* [1], *Andreas Hofmann* [1], *Hartmut Gliemann* [2], *Alexander Welle* [2], *Irene Wacker* [3], *Rasmus R. Schröder* [3,4], *Len Ness* [5], *Veit Hagenmeyer* [1], *Ulrich Gengenbach* [1]

[1] Institute for Automation and Applied Informatics, Karlsruhe Institute of Technology, Hermann-von-Helmholtz-Platz 1, 76344 Eggenstein-Leopoldshafen, Germany

[2] Institute of Functional Interfaces and Karlsruhe Nano Micro Facility (KNMF), Karlsruhe Institute of Technology, Hermann-von-Helmholtz-Platz 1, 76344 Eggenstein-Leopoldshafen, Germany

[3] Centre for Advanced Materials, Ruprecht-Karls-Universität Heidelberg, Im Neuenheimer Feld 225, 69120 Heidelberg, Germany

[4] Cryo Electron Microscopy, Universitätsklinik Heidelberg, BioQuant, Im Neuenheimer Feld 267, 69120 Heidelberg, Germany

[5] RMC Boeckeler, 4650 S. Butterfield Drive, Tucson, Arizona 85714, USA

***Corresponding Author**

Julian Hoffmann, Institute for Automation and Applied Informatics, Karlsruhe Institute of Technology, Hermann-von-Helmholtz-Platz 1, 76344 Eggenstein-Leopoldshafen, Germany

Email: julian.hoffmann@kit.edu

Supplementary Data S1: Dynamic ToF-SIMS analysis of a fresh sample

Prior to rinsing of the substrate the thickness difference for several siloxane fragments ($SiC_3H_9^+$ and $Si_2C_5H_{15}O^+$) comparing inside and outside of the channels is detected by a dynamic ToF-SIMS experiment for a 2 days old sample. The intensity ratio provides information about the relative siloxane layer height in exposed (left) and masked (right) areas of the substrate as shown in the Figure below.

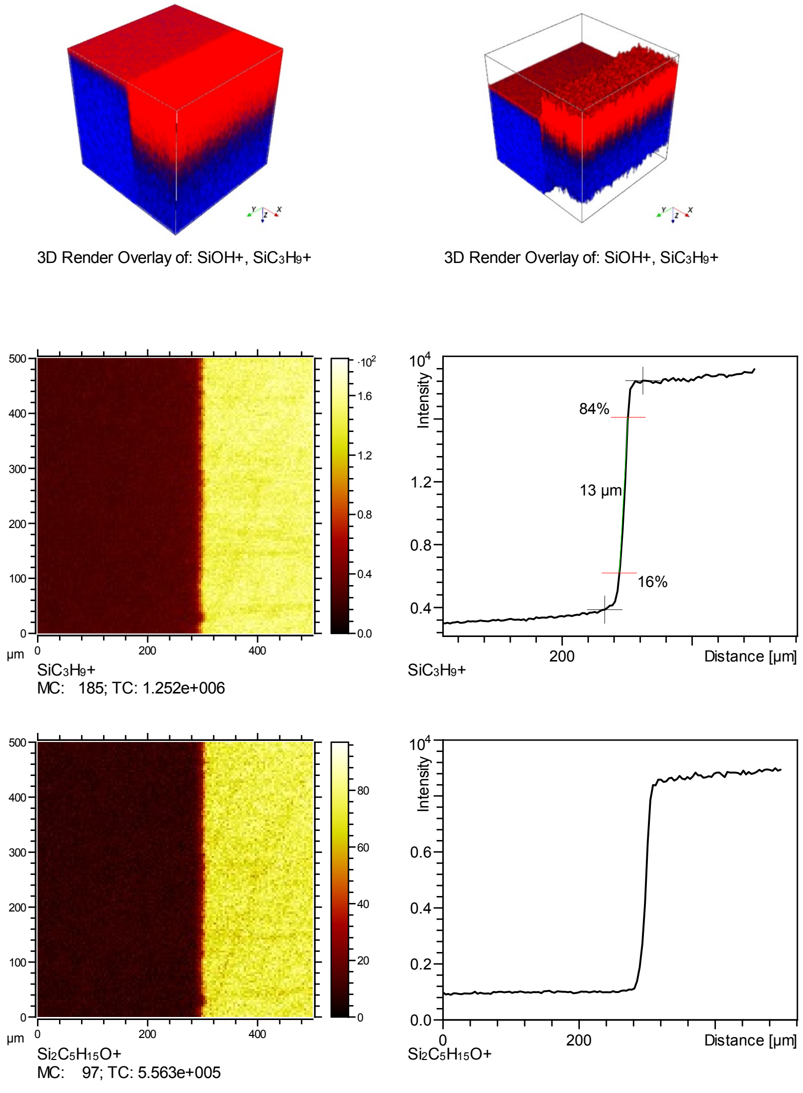

Figure for S1: Dynamic ToF-SIMS images of siloxane vapor coated glass after plasma treatment prior to rinsing. The heatmap images show a depth integrated signal. The topmost 2 pictures show a pseudo 3D rendering of raw data (left) and after shift correction for a flat glass surface indicated by $SiOH^+$ (blue). X=Y=500 µm. Z not to scale.

Supplementary Data S2: Static ToF-SIMS analysis with large field of view

To investigate the immobilization of the siloxane and possible PDMS residues from the applied stamp on the substrates, after 12 months of storage under ambient conditions a second set of ToF-SIMS data from the sample shown in the Figure below was recorded. Large areas search scans were performed to find a channel edge. Here, fast SIMS imaging is required, therefore high current bunched mode together with stage scanning is applied. The channel is highlighted by a strong sodium signal, potassium is found on some spots, together with $SiOH^+$ from the exposed glass. Several siloxane signals, lower row, show rather low contrast. This is due to several properties of the employed SIMS methodology:

- These signals are rather strong so detector saturation might occur on the left side of the imaged zone, showing the intact siloxane layer.
- The possible migration of low molecular weight siloxane fragments or residues from the applied PDMS stamp from the masked zone sidewards into the plasma exposed channel region. After a storage of 12 months for some fragments ($Si_2C_5H_{15}O$) this diffusion gradient is visible from left to right into the channel.
- Together with the low probing depth of SIMS (a few nanometer) already a thin layer of siloxane will yield a relatively strong signal in a static SIMS experiment.

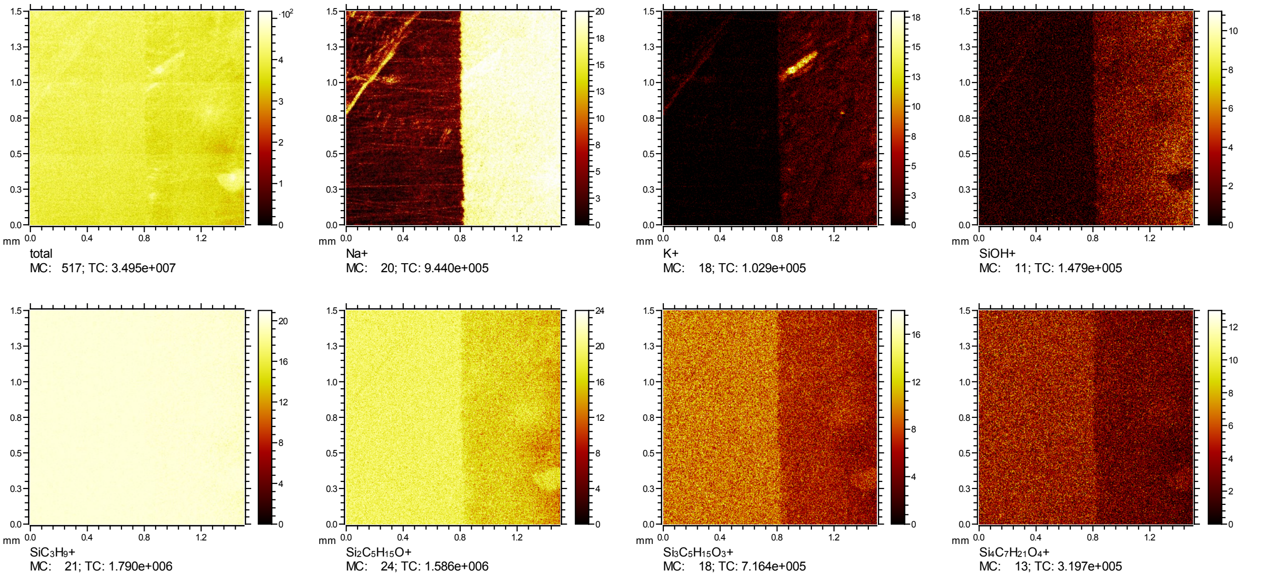

Figure for S2: Static ToF-SIMS images of siloxane vapor coated glass after plasma treatment and 12 months storage under ambient conditions and DI water rinsing. The channel is visible on the right.

Supplementary Data S3: Static ToF-SIMS analysis with reduced primary beam

A conventional imaging scan using high current bunched mode is performed. Scanning the primary beam, the field of view is limited to 500 µm × 500 µm as shown in the Figure below. By stepwise reducing the primary ion pulse width before bunching, the number of impingent primary ions per shot is reduced. This reduces the possibility of detector saturation. As shown in the Figure below (b), Reducing the primary beam to a quarter of the initial puls width, finally allows to see a weak contrast in the most abundant fragment SiC_3H_9, on the expense of a rather long data acquisition time.

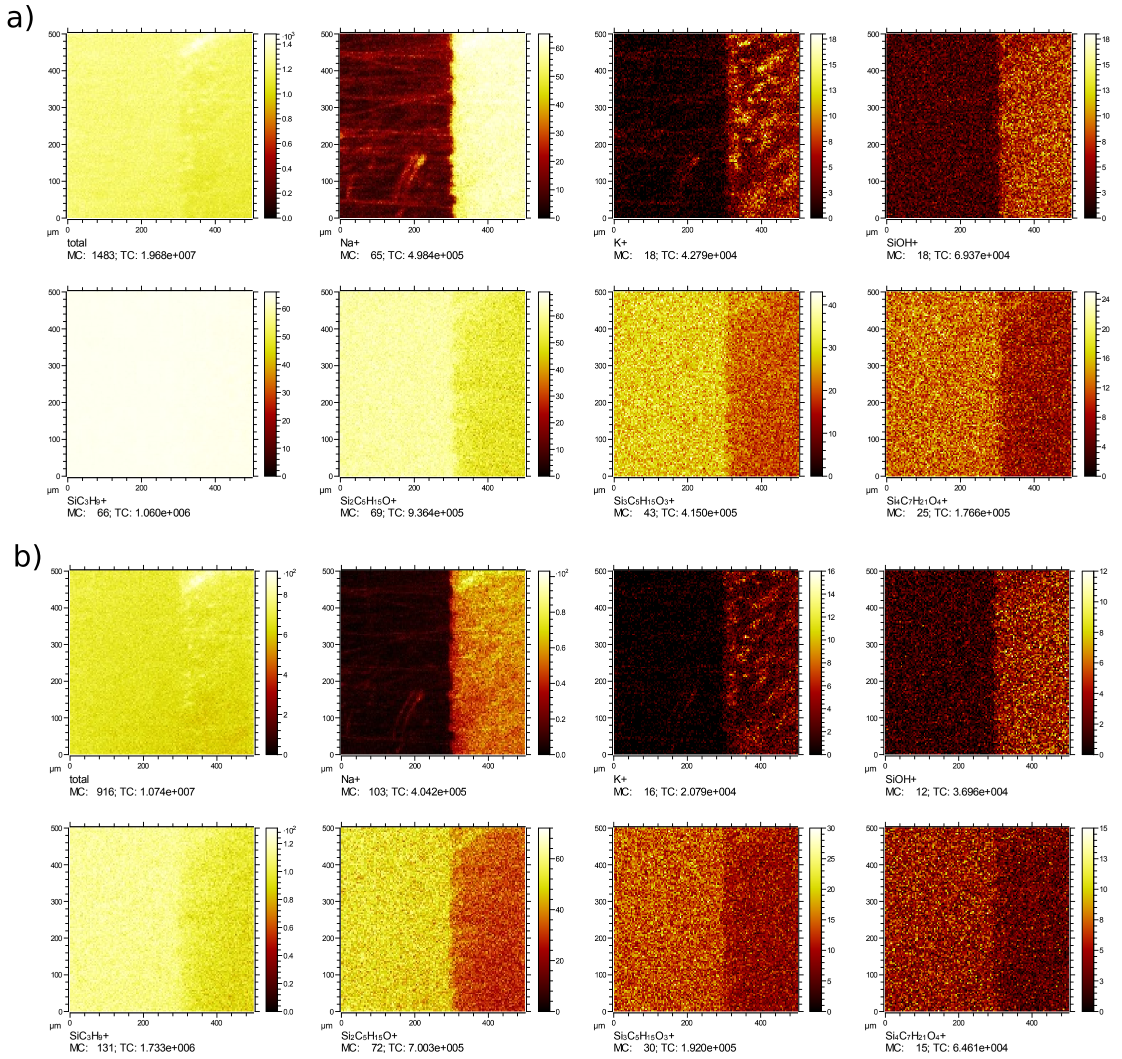

Figure for S3: Static ToF-SIMS images of siloxane vapor coated glass after plasma treatment and 12 months storage under ambient conditions and DI water rinsing. The channel is visible on the right. a) At full primary ion puls width and b) at quarter of the initial puls width.

Supplementary Data S4: Dynamic ToF-SIMS analysis of 12 months old sample

To demonstrate that also effects of migration and low probing depth are contributing to the low intensity ration in static measurement, an erosion experiment is performed. Therefore, in addition to the Bi_3^+ beam used for spectra recording, an argon cluster beam, Ar_{1300}, 5 keV, approx. 1 nA, was used to erode the sample (dynamic SIMS). The images shown below represent depth integrated lateral distributions. The full siloxane layer was eroded and probed, the image contrast for the siloxane signals can be interpreted as a thickness ratio. The intensity ratio between plasma exposed and masked areas is higher compared to the static measurements in Supplementary Data S3.

The width of the border (in other words the pattern fidelity) can be assessed by determining the width of the transition zone defined by two points (usually at 84% respectively 16% of the signal height (counts)). The width as shown in the Figure below is determined to be 14 µm. The relative siloxane layer height (1:4) is in good agreement to the measurements performed two days after sample preparation as seen in Supplementary Data S1. We therefore assume that the siloxane layer is stable.

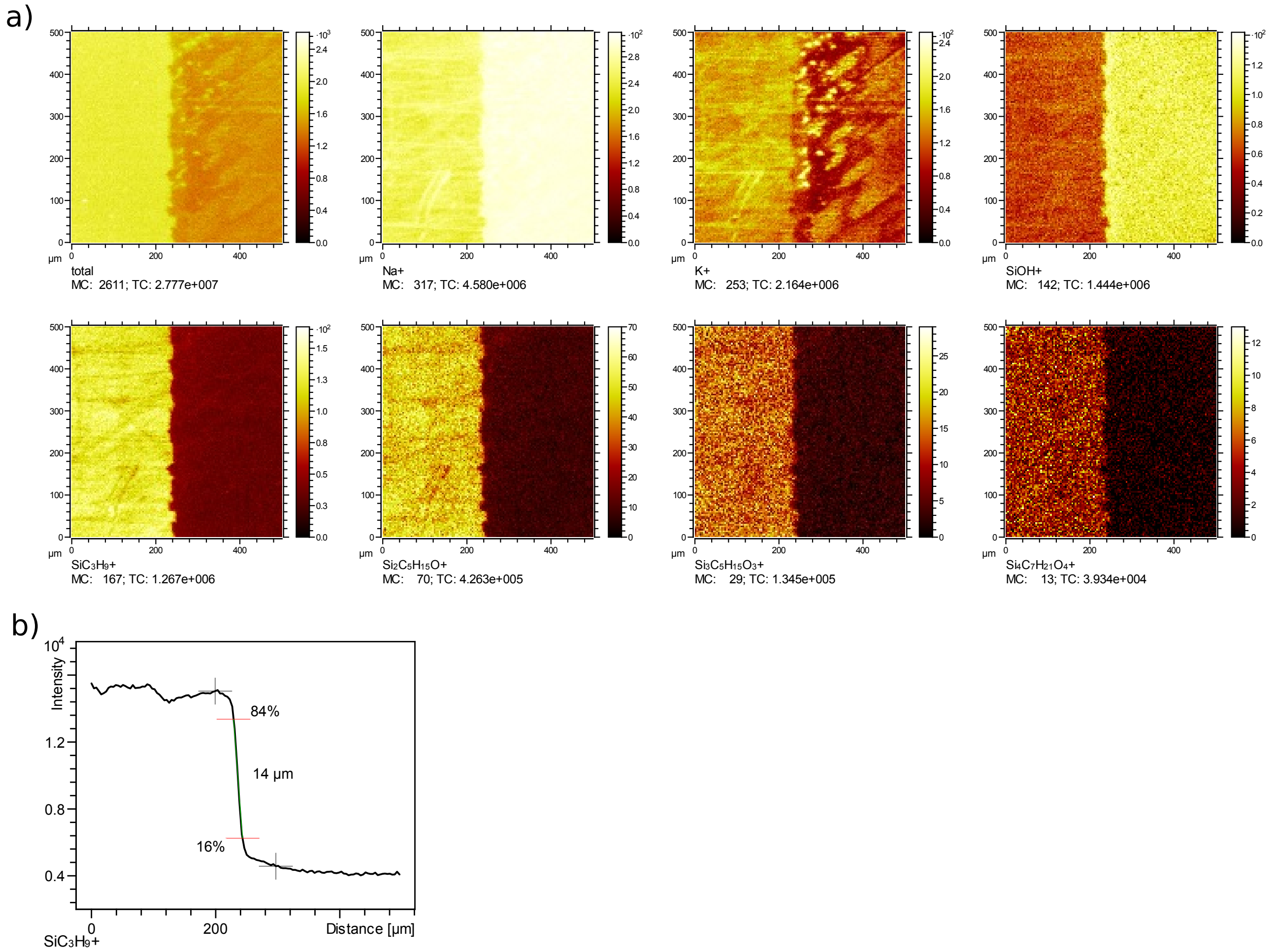

Figure for S4: a) Depth integrated dynamic ToF-SIMS images of siloxane vapor coated glass after plasma treatment and 12 months storage under ambient conditions and DI water rinsing. The channel is visible on the right. b) Intensity line profile of depth integrated dynamic ToF-SIMS image summed over 0.5 mm length along the channel edge.

Supplementary Data S5: Section rotation

Dimensions

w_1 : 0.85 mm

w_2 : 0.95 mm

h : 1.4 mm

b : 1.2 mm

$$\alpha_r = \alpha_1 + \alpha_2 - \alpha_3, \; with \; \alpha_1 = \sin^{-1}(h/d), \alpha_2 = \tan^{-1}\left(\frac{w_2 - w_1}{2h}\right), \alpha_3 = \cos^{-1}(b/d)$$

Maximum rotation (α_r) : 15.45°

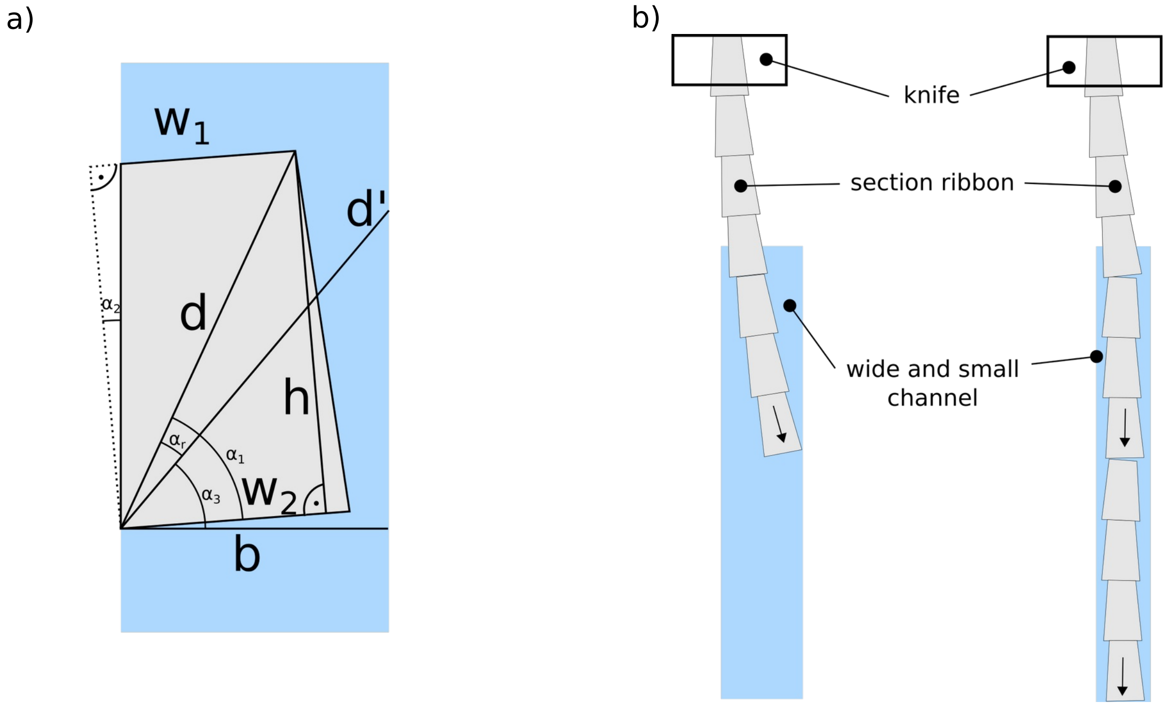

Figure for S5: a) Sketch of the maximum rotation α_r of trapezoidal sections with widths w_1 and w_2 and length h, between two straight boarders with distance b. b) Sketch of non tangential angles of contact between section and channel border, for wide and small channels.

Supplementary Data S6: Device for PDMS stamp positioning

Image of the device used for PDMS stamp positioning. The substrate is placed on the lower part aligned to the reference pins, the PDMS stamp is attached to the movable upper plate and presses onto the substrate. The whole assembly is placed in the plasma chamber.

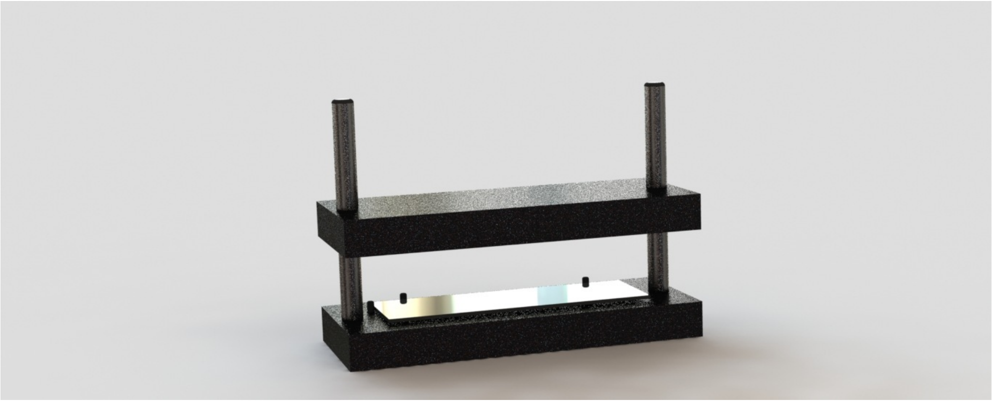

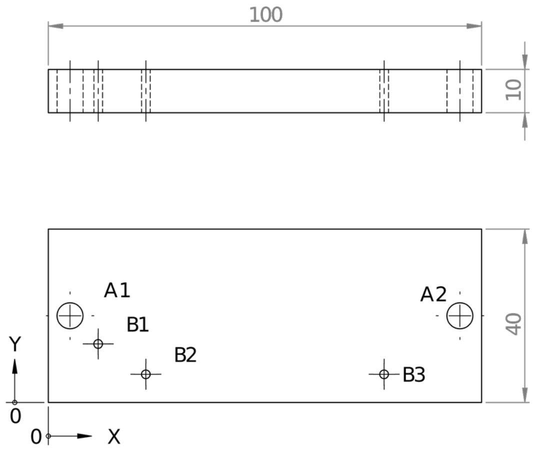

TAG	X LOC	Y LOC	SIZE
A1	5	20	∅ 6 THRU
A2	95	20	∅ 6 THRU
B1	11.50	13.50	∅ 2 THRU
B2	22.50	6.50	∅ 2 THRU
B3	77.50	6.50	∅ 2 THRU

Supplementary Data S7: Defect inspection of siloxane vapor coated glass at 9 different spots

ToF-SIMS images of siloxane vapor coated glass, defect inspection measurement (spots 1 to 9) in burst alignment mode, 512×512 pixel on 100×100 µm² with 100 scans, no defects found

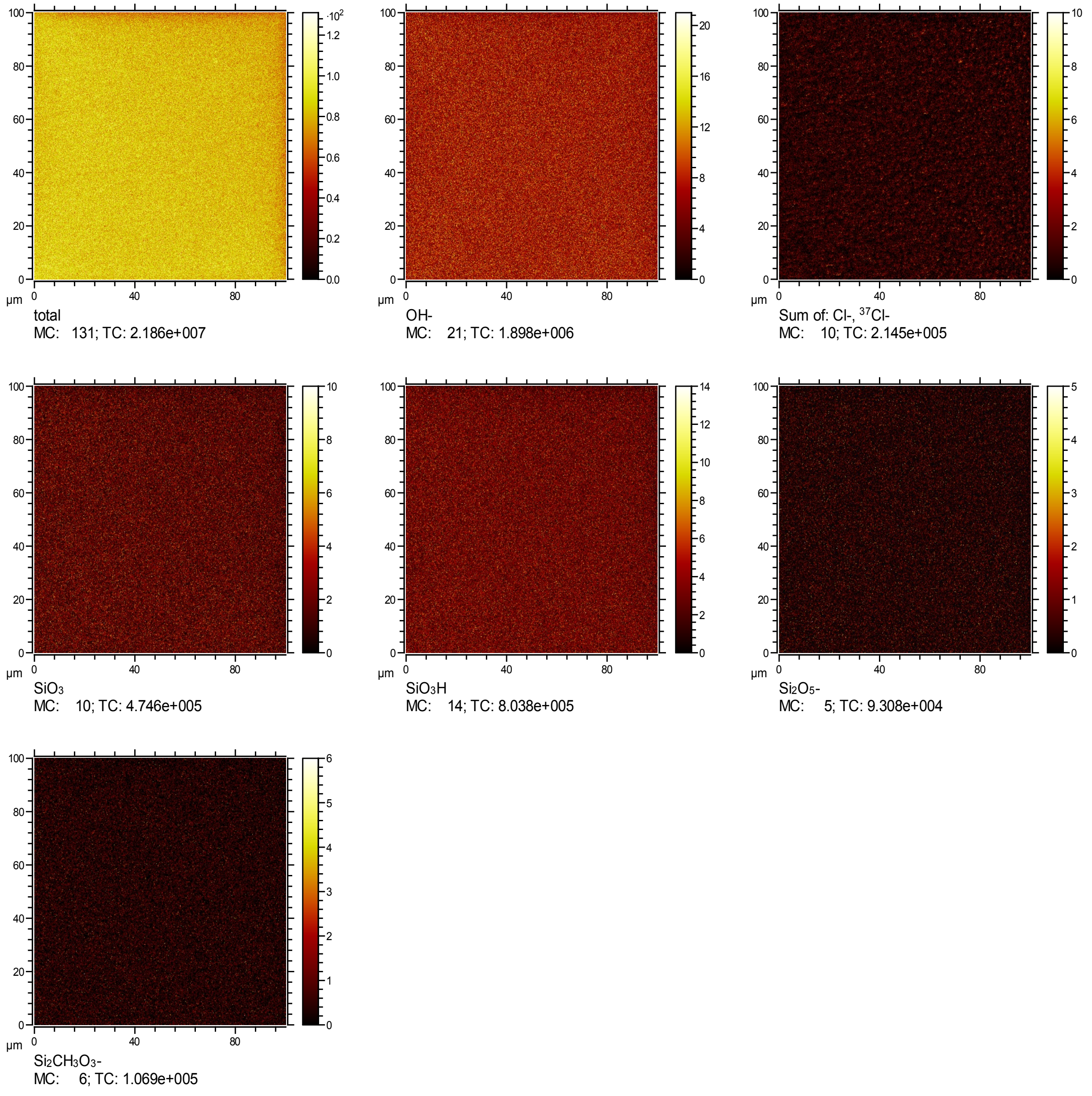

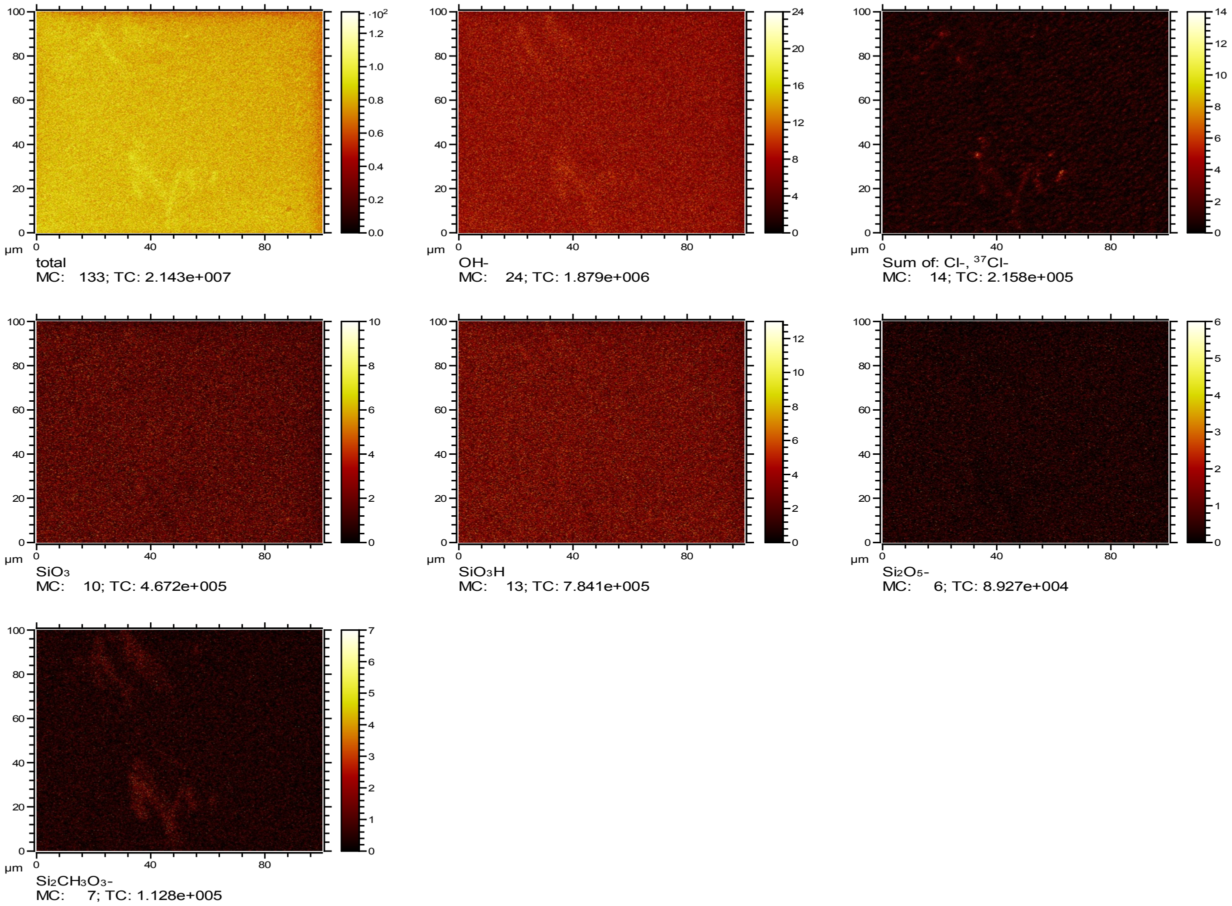

total
MC: 133; TC: 2.143e+007
OH-
MC: 24; TC: 1.879e+006
Sum of: Cl-, 37Cl-
MC: 14; TC: 2.158e+005
SiO3
MC: 10; TC: 4.672e+005
SiO3H
MC: 13; TC: 7.841e+005
Si2O5-
MC: 6; TC: 8.927e+004
Si2CH3O3-
MC: 7; TC: 1.128e+005
µm

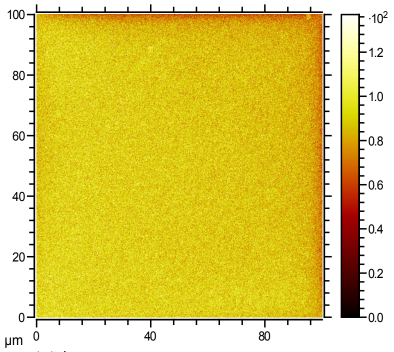

total
MC: 137; TC: 2.344e+007

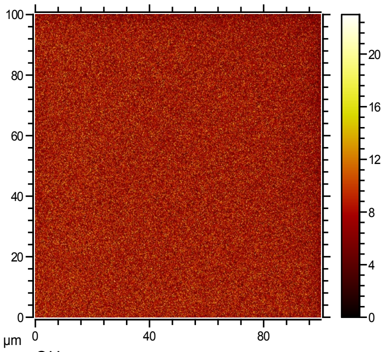

OH-
MC: 23; TC: 2.060e+006

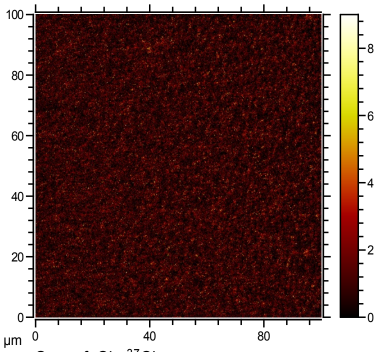

Sum of: Cl-, ^{37}Cl-
MC: 9; TC: 2.351e+005

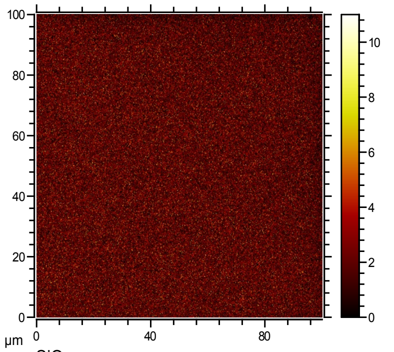

SiO_3
MC: 11; TC: 5.171e+005

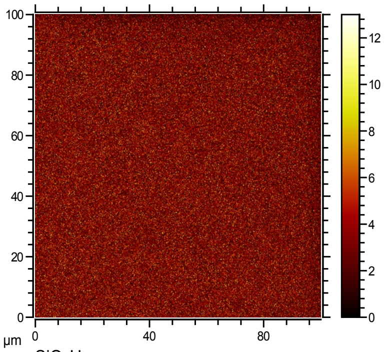

SiO_3H
MC: 13; TC: 8.553e+005

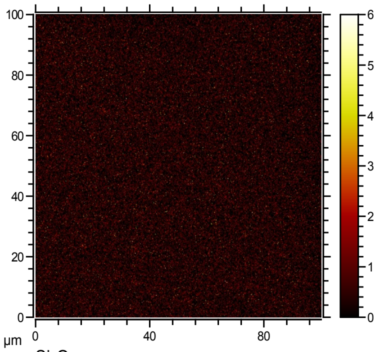

Si_2O_5-
MC: 6; TC: 1.006e+005

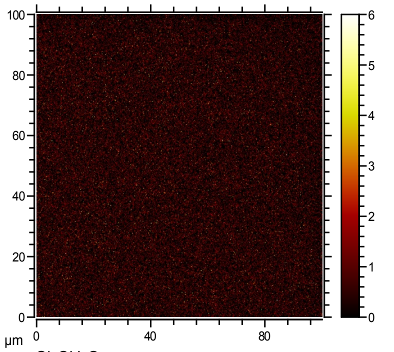

$Si_2CH_3O_3$-
MC: 6; TC: 1.139e+005

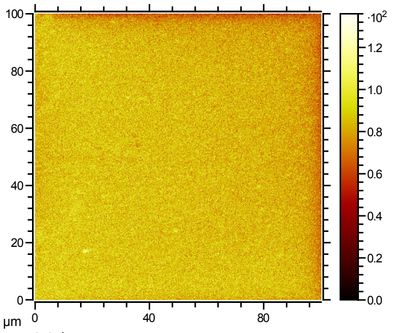

total
MC: 136; TC: 2.234e+007

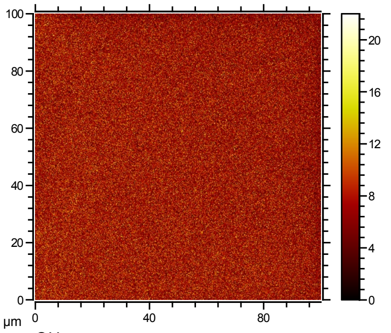

OH-
MC: 22; TC: 1.953e+006

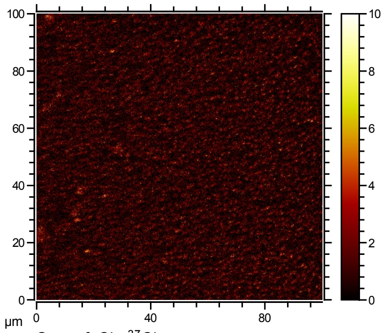

Sum of: Cl-, ^{37}Cl-
MC: 10; TC: 2.287e+005

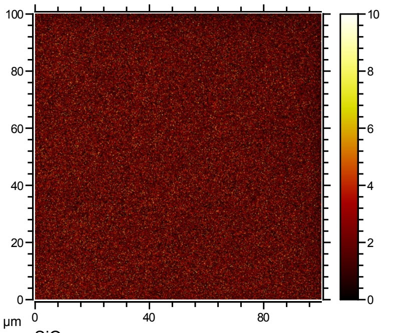

SiO_3
MC: 10; TC: 4.893e+005

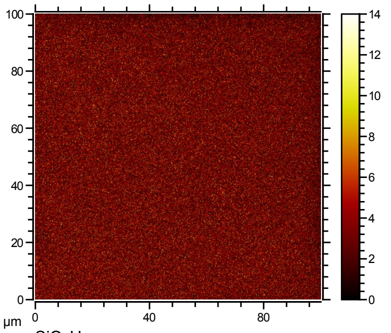

SiO_3H
MC: 14; TC: 8.122e+005

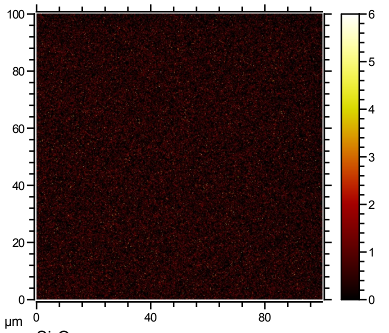

Si_2O_5-
MC: 6; TC: 9.504e+004

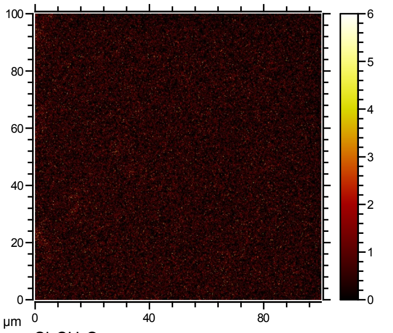

$Si_2CH_3O_3$-
MC: 6; TC: 1.081e+005

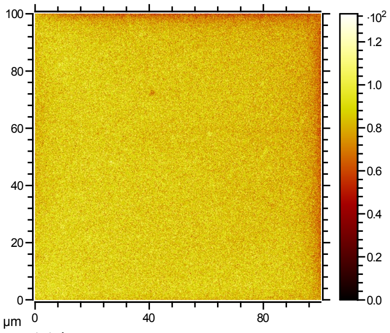

total
MC: 133; TC: 2.249e+007

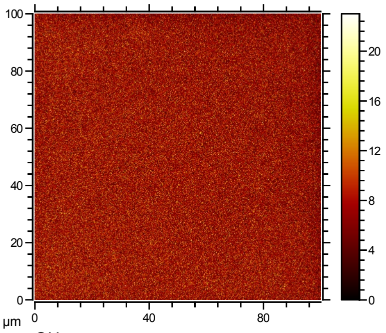

OH-
MC: 23; TC: 1.977e+006

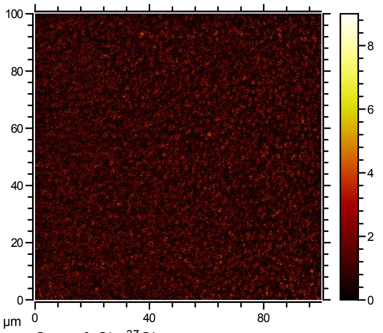

Sum of: Cl-, ^{37}Cl-
MC: 9; TC: 2.297e+005

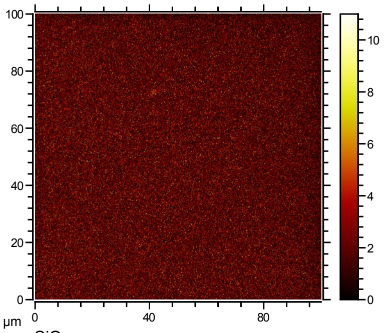

SiO_3
MC: 11; TC: 4.954e+005

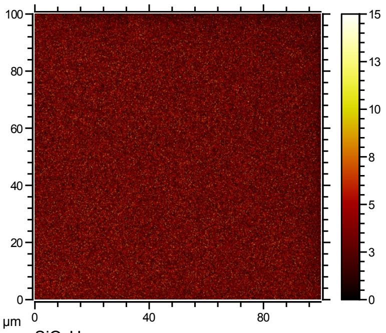

SiO_3H
MC: 15; TC: 8.174e+005

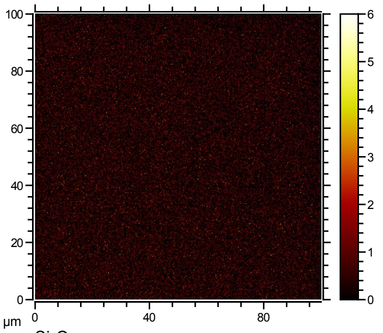

Si_2O_5-
MC: 6; TC: 9.608e+004

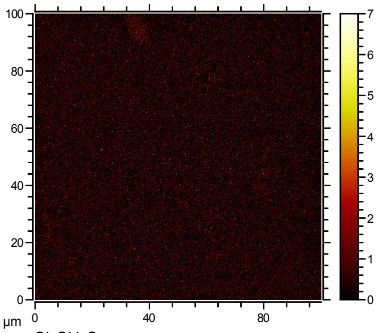

$Si_2CH_3O_3$-
MC: 7; TC: 1.076e+005

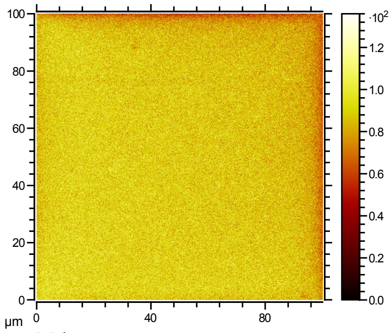

total
MC: 136; TC: 2.321e+007

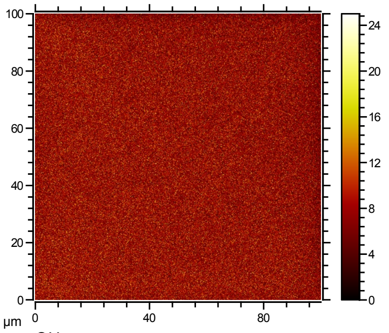

OH-
MC: 25; TC: 2.043e+006

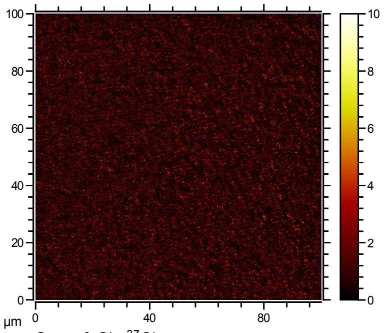

Sum of: Cl-, ^{37}Cl-
MC: 10; TC: 2.368e+005

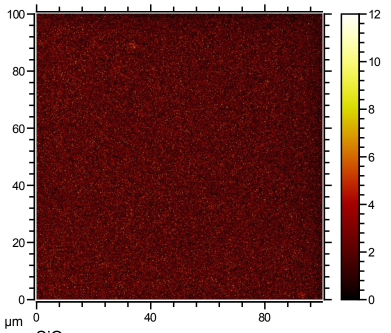

SiO_3
MC: 12; TC: 5.116e+005

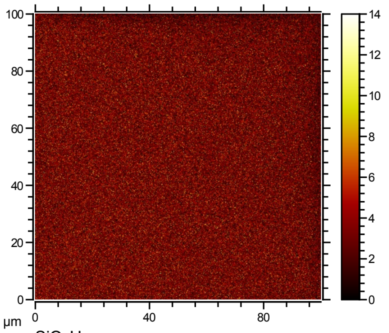

SiO_3H
MC: 14; TC: 8.399e+005

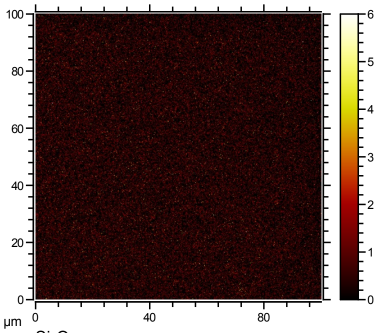

Si_2O_5-
MC: 6; TC: 9.870e+004

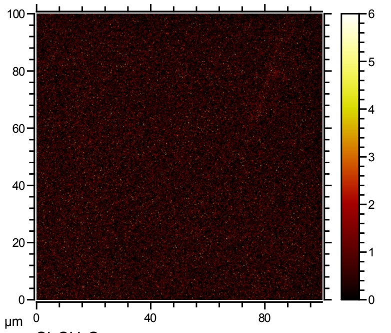

$Si_2CH_3O_3$-
MC: 6; TC: 1.094e+005

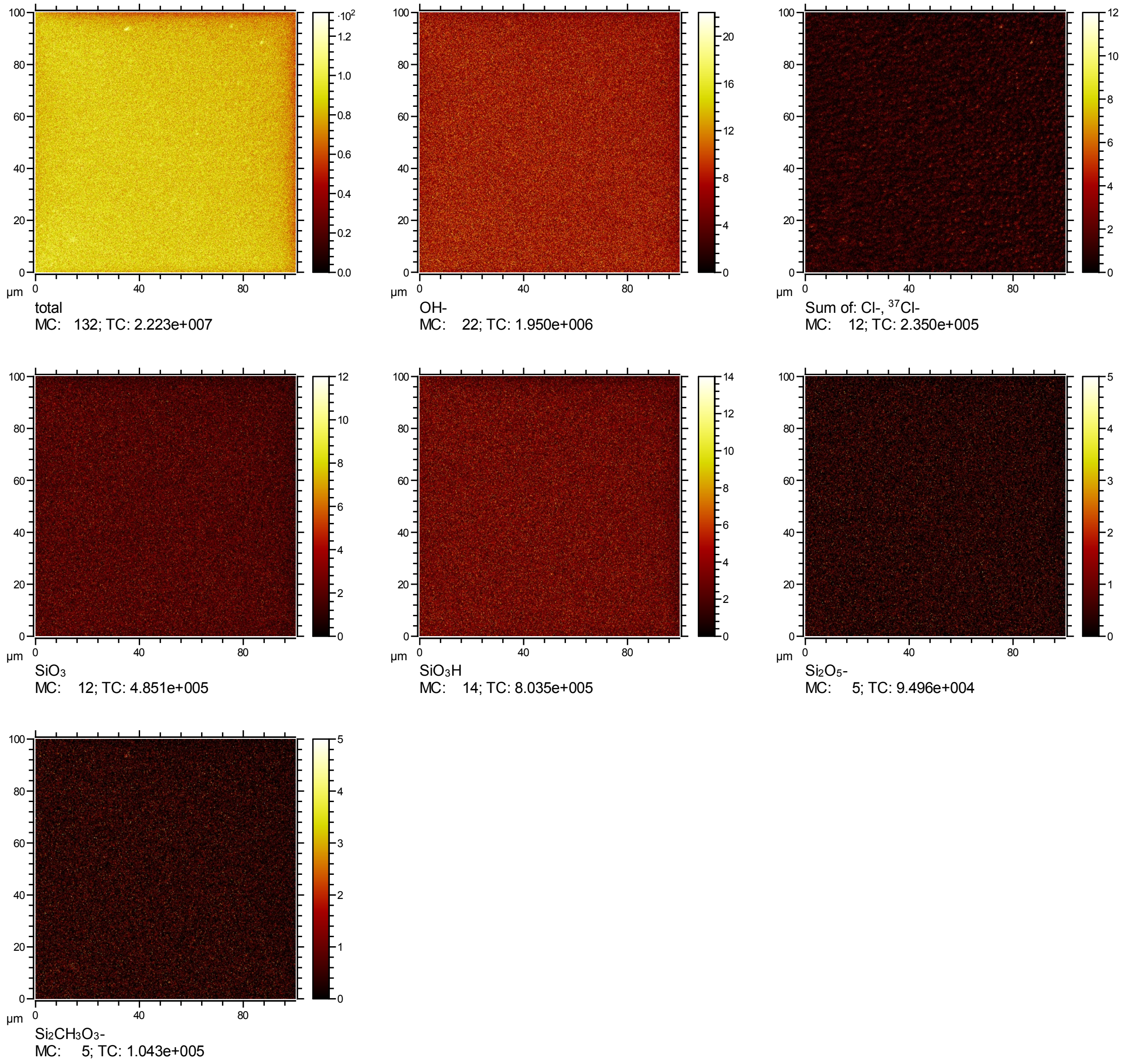
total
MC: 132; TC: 2.223e+007
OH-
MC: 22; TC: 1.950e+006
Sum of: Cl-, 37Cl-
MC: 12; TC: 2.350e+005
SiO3
MC: 12; TC: 4.851e+005
SiO3H
MC: 14; TC: 8.035e+005
Si2O5-
MC: 5; TC: 9.496e+004
Si2CH3O3-
MC: 5; TC: 1.043e+005
µm

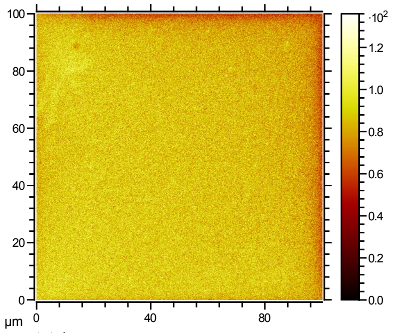

total
MC: 136; TC: 2.274e+007

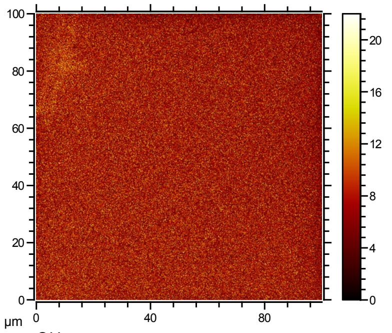

OH-
MC: 22; TC: 2.006e+006

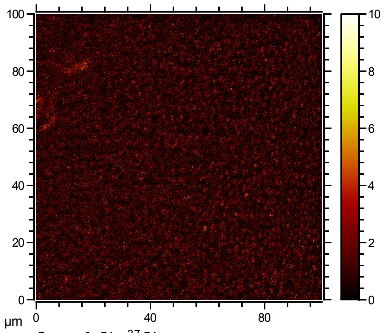

Sum of: Cl-, ^{37}Cl-
MC: 10; TC: 2.417e+005

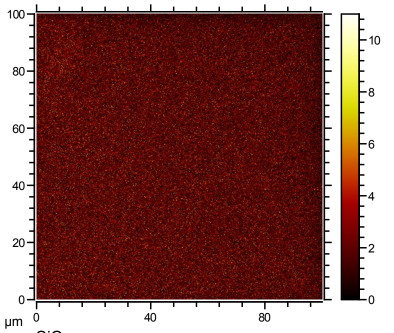

SiO_3
MC: 11; TC: 5.023e+005

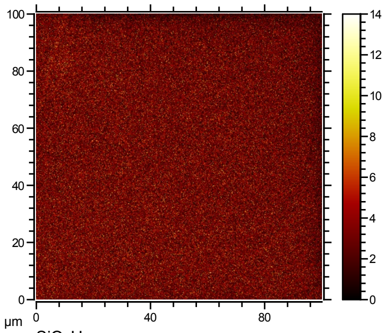

SiO_3H
MC: 14; TC: 8.175e+005

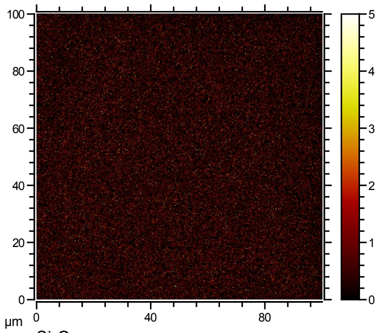

Si_2O_5-
MC: 5; TC: 9.643e+004

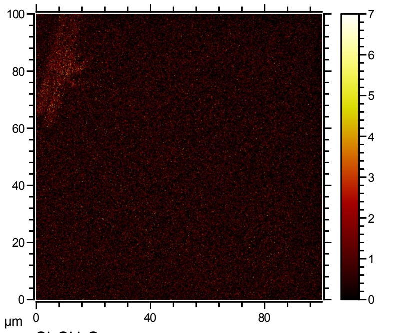

$Si_2CH_3O_3$-
MC: 7; TC: 1.094e+005

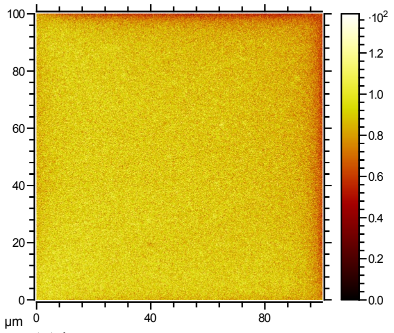

total
MC: 139; TC: 2.332e+007

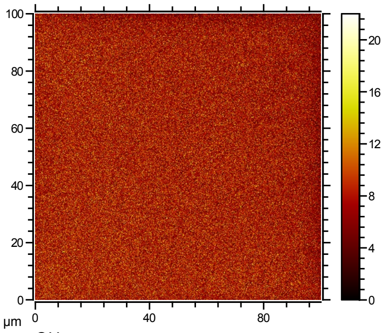

OH-
MC: 22; TC: 2.055e+006

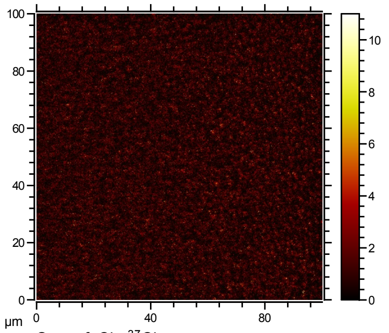

Sum of: Cl-, ^{37}Cl-
MC: 11; TC: 2.471e+005

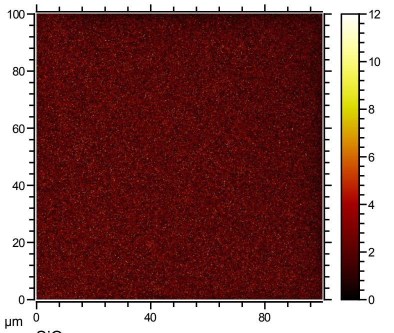

SiO_3
MC: 12; TC: 5.158e+005

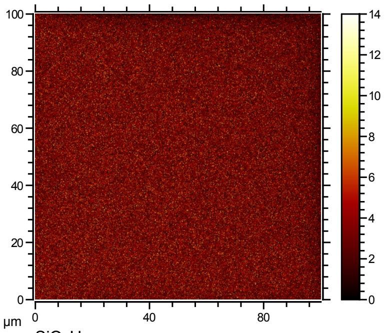

SiO_3H
MC: 14; TC: 8.349e+005

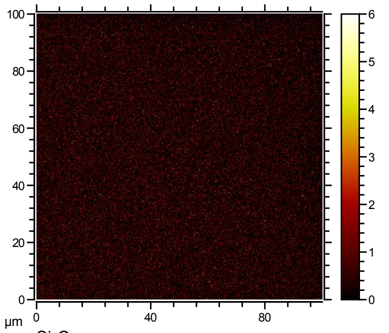

Si_2O_5-
MC: 6; TC: 9.956e+004

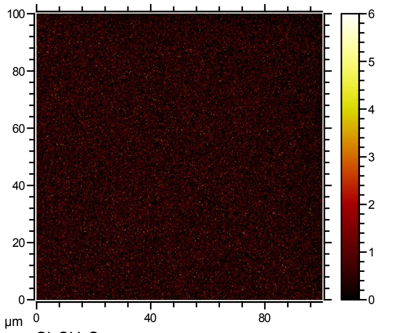

$Si_2CH_3O_3$-
MC: 6; TC: 1.074e+005

Legend for Video S8: Supplementary movie illustrating sliding of sections into the deposition template

Initial frame shows template with the first two channels already filled with sections. The pyramid-shaped resin block is seen at the top of the frame, opposite the knife edge.

The movie starts with movement of the substrate from channel 2 to the next empty channel. Then sectioning starts with the resin block moving and passing the knife edge, initially producing one section whose thickness and color is different from the following sections when cutting is in equilibrium stage. Even long ribbons are sliding easily into the channel – only 9 sections are shown in the course of this movie sequence.
